# The immunogenicity of plant-based COE-GCN4pII protein in pigs against the highly virulent porcine epidemic diarrhea virus strain from genotype 2

**DOI:** 10.3389/fvets.2022.940395

**Published:** 2022-07-28

**Authors:** Thuong Thi Ho, Vy Thai Trinh, Hanh Xuan Tran, Phuong Thu Thi Le, Tra Thi Nguyen, Hang Thu Thi Hoang, Minh Dinh Pham, Udo Conrad, Ngoc Bich Pham, Ha Hoang Chu

**Affiliations:** ^1^Graduate University of Science and Technology, Vietnam Academy of Science and Technology, Hanoi, Vietnam; ^2^Institute of Biotechnology, Vietnam Academy of Science and Technology, Hanoi, Vietnam; ^3^JSC Central Veterinary NAVETCO, Ho Chi Minh, Vietnam; ^4^Department Molecular Genetics, Leibniz Institute of Plant Genetics and Crop Plant Research (IPK), Gatersleben, Germany

**Keywords:** COE, GCN4-pII motif, PEDV genotype 2, plant-based vaccine, piglet protection, passive immunity

## Abstract

Porcine epidemic diarrhea virus (PEDV) is a serious infectious causative agent in swine, especially in neonatal piglets. PEDV genotype 2 (G2) strains, particularly G2a, were the primary causes of porcine epidemic diarrhea (PED) outbreaks in Vietnam. Here, we produced a plant-based CO-26K-equivalent epitope (COE) variant from a Vietnamese highly virulent PEDV strain belonging to genotype 2a (COE/G2a) and evaluated the protective efficacy of COE/G2a-GCN4pII protein (COE/G2a-pII) in piglets against the highly virulent PEDV G2a strain following passive immunity. The 5-day-old piglets had high levels of PEDV-specific IgG antibodies, COE-IgA specific antibodies, neutralizing antibodies, and IFN-γ responses. After virulent challenge experiments, all of these piglets survived and had normal clinical symptoms, no watery diarrhea in feces, and an increase in their body weight, while all of the negative control piglets died. These results suggest that the COE/G2a-pII protein produced in plants can be developed as a promising vaccine candidate to protect piglets against PEDV G2a infection in Vietnam.

## Introduction

Porcine epidemic diarrhea (PED) is an acute and highly contagious enteric disease in swine, caused by the porcine epidemic diarrhea virus (PEDV) (1). Pigs of all ages can be infected by PEDV, resulting in the rapid spread of acute and severe diarrhea, dehydration, and vomiting. Suckling piglets, especially at the age of under 7 days, are the most severely affected with mortality reaching nearly 100% (2). Since the first outbreaks in Europe, PEDV has caused severe economic loss to the pig industry worldwide, particularly in Asian countries and the United States (1, 3, 4). In Vietnam, PED was first reported in 2009 and then continuously spread in several pig farms throughout the country, causing remarkable economic damage (5).

Porcine epidemic diarrhea virus is a single-stranded RNA virus and a member of the genus Alphacoronavirus, in the family Coronaviridae (6). PEDV can be clustered into two genogroups (G1 and G2) based on the nucleotide and amino acid sequence of Spike (S), especially the changes in amino acid sequence at the N-terminal of the S1 domain (7, 8). Each genogroup contains subgroups a and b. PEDV strains circulating in the north, central, and southern regions of Vietnam from 2015 to 2016 belonged to G1b, G2a, and G2b (4, 9). The differentiation in genetic information between the emerging PEDV strains and PEDV vaccine strains, especially at the neutralizing epitopes, results in the low effectiveness of the current vaccine in the protection of pigs against PEDV strains in the field (9–11). In Vietnam, PEDV G2 strains were found as the major disease causative agents in PED outbreaks (4). To date, subunit vaccines against Vietnamese PEDV G2 strains are not available. Therefore, the development of a novel vaccine against emerging PEDV strains, especially G2 strains, is urgent to control PED outbreaks in Vietnam (4, 9).

The CO-26K-equivalent epitope (COE) is located in amino acids 499–638 within the Spike (S) protein (12). It is a neutralizing epitope recognized by the monoclonal antibody (6). COE protein is considered an important target for developing subunit vaccines against PEDV (12, 13). The plant-based production systems, especially agroinfiltration, have been widely considered a rapid, convenient, and low-cost method to produce a large number of recombinant proteins within a few days in several plants (14, 15). There have been efforts to produce S1 or COE protein as fusion forms against PEDV in various plants such as lettuce, rice, *Nicotiana benthamiana, Nicotiana tabacum*, and maize using stable transformation or transient expression (13, 16–21). In a previous study, we demonstrated that plant-based COE/G1a-pII induced neutralizing antibodies in mice (22). However, the immunogenicity and protectivity of plant-based COE/G1a-pII in piglets against PEDV *via* challenge experiments have still not been evaluated.

This study aims to evaluate whether our previous concept could also be worked on another COE variant from a highly virulent PEDV strain belonging to G2 (COE/G2a-pII), which is the currently circulating strain in Vietnam. Moreover, we analyzed if a two-dose vaccination scheme with COE/G2a-pII could induce protective immune responses in piglets from virulent PEDV strains *via* challenge experiments. As expected, all piglets from sows vaccinated with purified COE/G2a-pII protein survived, while all negative control piglets died after the challenge with the highly virulent PEDV G2a strain. In addition, COE/G2a-pII elicited high levels of neutralizing antibodies, PEDV-specific IgG antibodies, COE-specific IgA antibodies, and IFN-γ in sows and piglets.

## Materials and methods

### Production and characterization of COE/G2a-PII protein from plants

A DNA sequence encoding for the COE/G2a region from the highly virulent NAVET/PEDV/PS6/2010 strain (NAVETCO, Vietnam) belonging to G2a was codon-optimized for expression in *N. benthamiana*, synthesized, and inserted into a cloning vector pUC57-COE (Genewiz, United States). COE/G2a was amplified and inserted in pRTRA-COE/G1a-GCN4pII-cmyc-his-KDEL (22) at sites *Bam*HI and *psp*OMI. A plant expression vector containing COE/G2a-pII was then constructed according to the previous study (22). In this study, COE/G2a-pII protein was expressed in *N. benthamiana via* the agroinfiltration protocol that was mentioned in the previous study (22) with a modification in the OD_600_ agrobacterium suspension mixture of 0.1. After 3 days of infiltration, leaves were collected and stored at −80°C. The expression of COE/G2a-pII protein in plants was detected by SDS-PAGE and Western blot assay using anti-cmyc-antibody as the primary antibody and goat anti-mouse IgG-HRP (Invitrogen) as the secondary antibody. A signal was detected using the Amersham ECL Prime Western blotting detection reagent. The expression level of COE/G2a-pII protein was measured based on the H5N1-specific ScFv protein amount in a standard curve (23) that was conducted and analyzed using the ImageQuant TL 8.0 software (Cytiva) after the signal was detected using the Amersham™ Imager 680 machine. Leaves were subjected to COE/G2a-pII protein purification using the immobilized metal affinity chromatography (IMAC) protocol that was reported in the previous study with a modification in washing buffer containing 10 mM of imidazole (22).

The molecular weight of COE/G2a-pII protein was characterized by size exclusion chromatography (SEC) following the protocol as described earlier (24). An amount of 500 μg IMAC-purified COE/G2a-pII protein was applied onto a Superose™ 6 increase 10/300GL column (GE Healthcare). A high molecular weight kit (GE Healthcare) containing standard proteins with molecular weights ranging from 75 to 2,000 kDa was used to estimate the molecular weight of COE/G2a-pII protein. The presence of COE/G2a-pII protein in SEC fractions was confirmed by SDS-PAGE and Western blotting assay.

### Cells and viruses

Vero cells (ATCC CCL-81) were cultured in Eagle's Minimum Essential Medium (EMEM, Gibco) supplemented with 10% fetal bovine serum (FBS, Gibco), 50 μg/ml gentamycin at 37°C, and 5% CO_2_. The NAVET/PEDV/PS6/2010 strain was isolated from piglet samples infected with PEDV and propagated on Vero cells using the protocol previously described (25). The virus in passage 5 was used for challenge experiments in piglets. There was no change in the nucleotide sequence of the S gene of the NAVET/PEDV/PS6/2010 strain in passage 5 when compared to that of the isolated PEDV strain.

### Pig immunization and viral challenge experiments

The pig study was reviewed and approved by the ethical committees of the Institute of Biotechnology (IBT); Vietnam Academic of Science and Technology (VAST), Hanoi, Vietnam; and NAVETCO, Ho Chi Minh City, Vietnam, under decision number 07/2015/HÐ-NÐT. The animal protocols were performed based on the “3Rs” and the European Communities Council Directives of 24 November 1986 (86/609/EEC) guidelines for the care and use of animals. Pigs were raised by NAVETCO, Vietnam, and monitored to minimize the stress, suffering, unnecessary pain, or lasting harm in the experiments by veterinarians.

Before 2 weeks of vaccination, blood samples of pregnant sows were collected for serum neutralizing antibody assays performed at NAVETCO's laboratory. Two pregnant sows with negative results of neutralizing antibodies against PEDV were selected for the vaccination experiments. Either purified COE/G2a-pII protein or PBS was formulated extensively with in-house water in oil adjuvant (NAVETCO, Vietnam) at a ratio of 3:7. The primiparous sow (~80 days of gestation, *n* = 1) was intramuscularly immunized on the neck with either adjuvanted COE/G2a-pII protein (100 μg per dose) or PBS on days 0 and 14 post-immunization (pi). After 21 days of the second immunization, farrowing was induced. Sow blood samples were collected on days 0, 35, and 50 pi. Eight piglets born to one COE/G2a-pII-vaccinated sow and two piglets born to one PBS-vaccinated sow were suckled and kept together with their sows. Each 5-day-old piglet was challenged orally with 1 ml of the highly virulent NAVET/PEDV/PS6/2010 strain (10^3^ TCID50). Piglets were monitored daily for clinical signs, diarrhea, weight, and death from days 0 to 14 post-challenge (pc). Clinical scores were modified from a scoring method previously described (26). Clinical scores were defined as follows: healthy = 0; anorectic, depression, vomiting, and emaciated = 1; dead = 2. The body weight of piglets was recorded on days 0 and 10 pc. Fecal scores were evaluated as follows based on a scoring method previously described (27): normal = 0, pasty = 1, semi-solid = 2, watery diarrhea = 3. Blood samples of all the piglets were collected *via* the marginal ear veins in serum separator tubes on days 0 and 10 pc for analysis. All blood samples were centrifuged at 3,000 × *g* for 10 min at 4°C, and the sera were stored at −20°C until analysis.

### ELISA

PEDV-specific IgG antibodies in the sera of sows and piglets were assessed using a commercial ELISA kit (INgezim PEDV 11.PED.K.1/5, Eurofins INGENASA) according to the manufacturer's recommendation. The S/P values were calculated using the formula: [(test sample value–negative control value) / (positive control value–negative control values)]. Samples were defined as positive for PEDV-specific IgG if the S/P value is > 0.35. COE-specific IgA antibodies in sow and piglet sera were analyzed using an indirect ELISA using SEC-purified COE/G2a-pII protein (1 μg/ml) as antigen according to the protocol described previously (22).

### Virus neutralizing antibody test

The presence of neutralizing antibodies in porcine sera was evaluated using a virus-neutralizing assay according to the protocol previously described (28). PEDV strain NAVET/PEDV/PS6/2010 (200 TCID50/0.1 ml) was performed for the assay. The virus neutralization titer (VN titer) was determined as the highest serum dilution that inhibited the cytopathic effect.

### Cytokine assay

The level of interferon-gamma (IFN-γ) in the sera of sows and piglets was detected using a commercial ELISA kit (Porcine IFN-γ ELISA Basis kit, Matech) according to the manufacturer's recommendation. Porcine sera with a dilution of 1:20 were used for the cytokine assay. The concentration of IFN-γ in diluted sera (IFN-γ DL) was calculated based on the standard curve of recombinant porcine IFN- γ standard with the range of 16–1,600 pg/ml. The final concentration of IFN-γ in pig sera was calculated using the formula: IFN-γ DL × 20.

### Statistical analysis

All statistical analyses were performed in the Sigma Plot software. The differences between the two groups were compared and shown as mean ± standard deviation (SD) using a Mann–Whitney U test. The significant difference was determined if the *p*-value was < 0.05.

## Results

### Production and characterization of COE/G2a-PII

**Figure 1:**
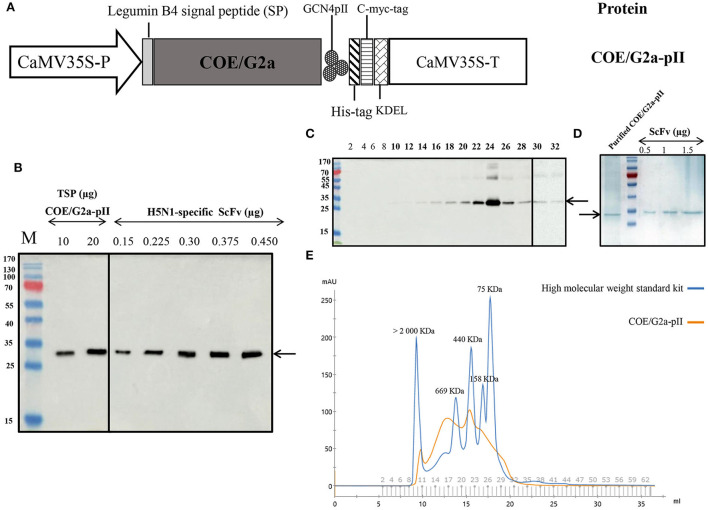
Production and characterization of COE/G2a-pII protein from plants. **(A)** Plant expression cassette containing a DNA sequence encoding the highly virulent NAVET/PEDV/PS6/2010 strain (NAVETCO, Vietnam) fused pII motif. CaMV35S-P, cauliflower mosaic virus (CaMV) 35 S promoter; pII, GCN4pII motif; KDEL, endoplasmic reticulum retention; CaMV35S-T, CaMV 35 S terminator. **(B)** Detection of COE/G2a protein expressed in *N. benthamiana* leaves by Western blotting assay. Anti-c-myc monoclonal antibody and HRP conjugated goat anti-mouse IgG were used as primary antibodies and secondary antibodies, respectively. Various amounts of H5N1-specific ScFv antibodies (23) were used to build the standard curve to calculate the accumulation of COE/G2a-pII protein in leaves by ImageQuant TL (Cytiva) after capturing by Amersham™ Imager 680 (Cytiva). **(C)** Detection of SEC-fractions containing COE/G2a-pII protein by Western blotting assay. **(D)** Analysis of IMAC-purified COE/G2a-pII protein by Coomassie Blue staining of SDS-PAGE. **(E)** Characterization of COE/G2a-pII protein by SEC. A standard kit including high molecular weight protein (75–2,000 kDa, GE) was used to estimate the molecular weight of COE/G2a-pII protein.

To construct the expression cassette harboring COE/G2a-pII, we replaced the DNA sequence encoding COE from the PEDV DR13 strain (G1a) with that encoding COE/G2a from the highly virulent NAVET/PEDV/PS6/2010 strain (Figure 1A). The expression of COE/G2a-pII protein in *N. benthamiana* leaves was successfully detected by a Western blotting test using anti-cmyc-antibody as the primary antibody (Figure 1B). The accumulation of COE/G2a-pII protein in leaves was calculated based on the standard curve of the H5N1-specific ScFv protein (23). COE/G2a-pII protein accumulated in plant leaves at approximately 118 mg/kg fresh leaves, which accounted for 2.01% of total soluble protein (TSP). COE/G2a-pII protein was purified by IMAC, and then the resulting product was further purified and characterized by SEC (Figures 1C,D). The predicted molecular weight of COE/G2a-pII monomer is approximately 26 kDa; however, N-glycosylation sites in COE protein may influence the PAGE separation resulting in the increase in band size of COE/G2a-pII protein in SDS-PAGE and Western blot of approximately 35 kDa (Figure 1D). The molecular weight of COE/G2a-pII was calculated and characterized by SEC based on the standard kit containing high molecular weight proteins (75 to >2,000 kDa). COE/G2a-pII protein was presented in SEC fractions 10–32 corresponding to the molecular weight of 75 to >2,000 kDa (Figure 1E). COE/G2a-pII protein was mostly detected in SEC fractions 22–26 corresponding to the molecular weight of approximately 440 kDa. Therefore, COE/G2a-pII is a mixture of multimer forms with a wide range in molecular weight.

### Humoral immune responses and cytokine responses in sows

**Figure 2:**
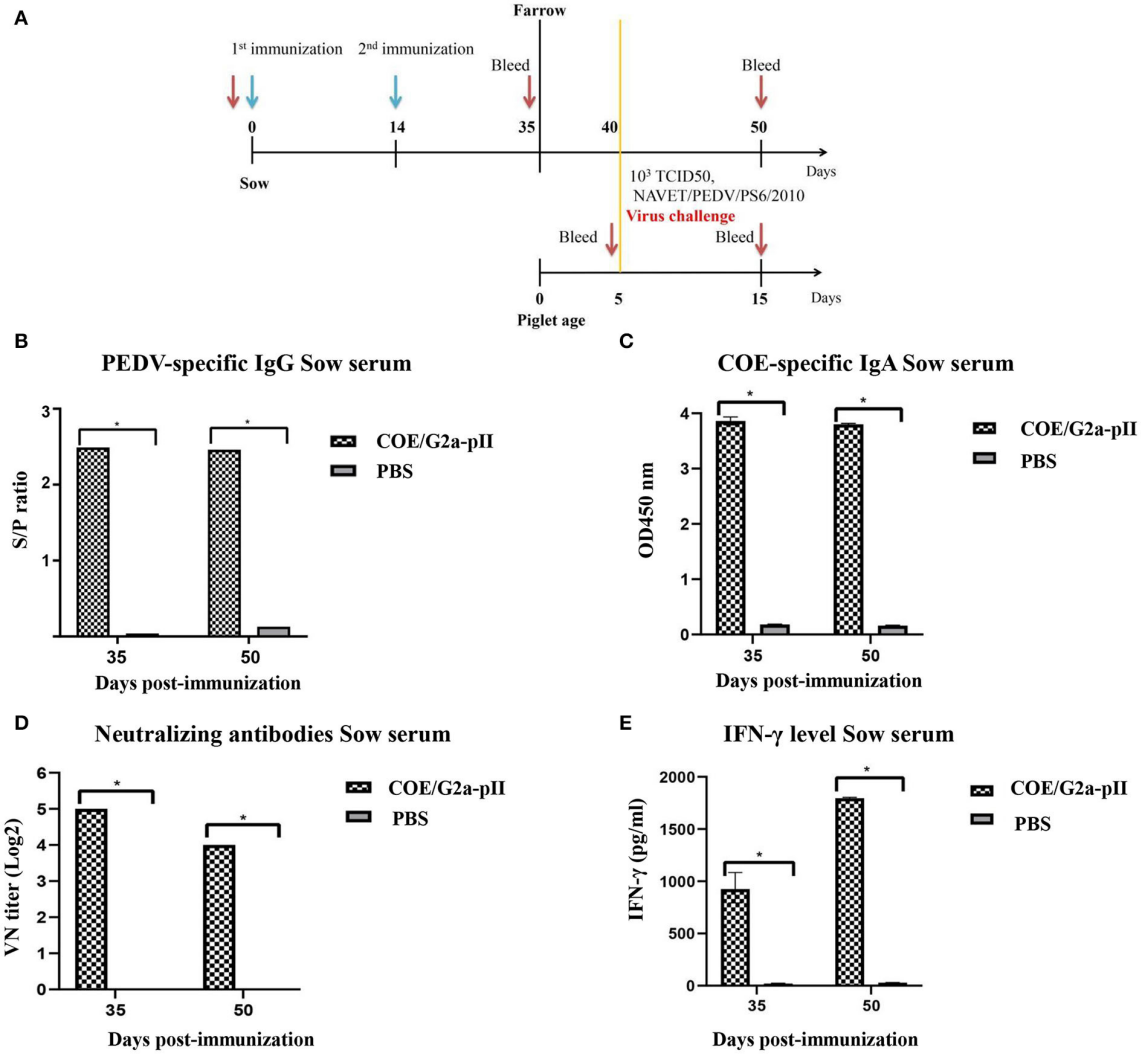
Humoral immune responses and cytokine responses in pregnant sows. Data are presented as mean ± standard deviation (SD). * indicates a statistically significant difference (*p* < 0.05). **(A)** Immunization and challenge experiment scheme in pregnant sows (~80 days of gestation, *n* = 1 per group) and piglets (*n* = 8 for COE/G2a-pII group and *n* = 2 for PBS group). The green arrow and red arrow indicate immunization and bleeding time, respectively. **(B)** PEDV-specific IgG antibodies in sow sera vaccinated with COE/G2a-pII or PBS were measured by a commercial ELISA kit. An S/P ratio > 0.35 was defined as positive with PEDV-specific IgG antibody. **(C)** COE-specific IgA antibodies in sow sera were evaluated by an ELISA using SEC-purified COE/G2a-pII as antigen. **(D)** Neutralizing antibodies in sow sera were analyzed by the virus-neutralizing assay. The highly virulent NAVET/PEDV/PS6/2010 strain (200 TCID50/0.1 ml) was used for the assay. A VN titer ≥ 8 was defined as positive with neutralizing antibody against PEDV. **(E)** IFN-γ levels in sow sera were measured based on the standard curve of recombinant porcine IFN- γ standard in a commercial ELISA kit.

The immunogenicity of IMAC-purified COE/G2a-pII protein was evaluated in pregnant sows. The immunization scheme in pregnant sows is presented in Figure 2A. PEDV-specific IgG responses were detected in the serum of the sow vaccinated with COE/G2a-pII protein with an S/P value of 2.49 on day 35 pi (Figure 2B). There was no increase in PEDV-specific IgG responses in this inoculated sow on day 50 pi compared to that on day 35 pi. In contrast, no PEDV-specific IgG antibody was found in the sow immunized with PBS. The pregnant sow vaccinated with COE/G2a-pII protein developed COE-specific IgA antibodies in serum on day 35 pi with an OD450 mean value of 3.8. Similar to the PEDV-specific IgG responses, the COE-specific IgA antibodies did not increase in the serum of this vaccinated sow on day 50 pi. In contrast, COE-specific IgA antibodies were not detected in the serum of the sow immunized with PBS (Figure 2C). The presence of neutralizing antibodies against PEDV in sow sera was assessed by virus-neutralizing antibody assay. On day 35 pi, the VN titer of 32 was observed in the sow vaccinated with COE/G2a-pII protein. However, the neutralizing antibody response of the COE/G2a-pII-immunized sow was decreased on day 50 pi (Figure 2D). The IFN-γ were found at a high concentration of 932.3 ± 145.2 pg/ml in serum of the sow injected with COE/G2a-pII protein on day 35 pi. Notably, there was a 2-fold increase in IFN-γ level (1796.1 ± 8.6 pg/ml) in this sow on day 50 pi (Figure 2E). IFN-γ was not found in the negative control sow vaccinated with PBS. These data indicate that the COE/G2a-pII protein induces strong humoral immune responses and cytokine responses in the pregnant sow.

### Humoral immune responses and cytokine responses in piglets

**Figure 3:**
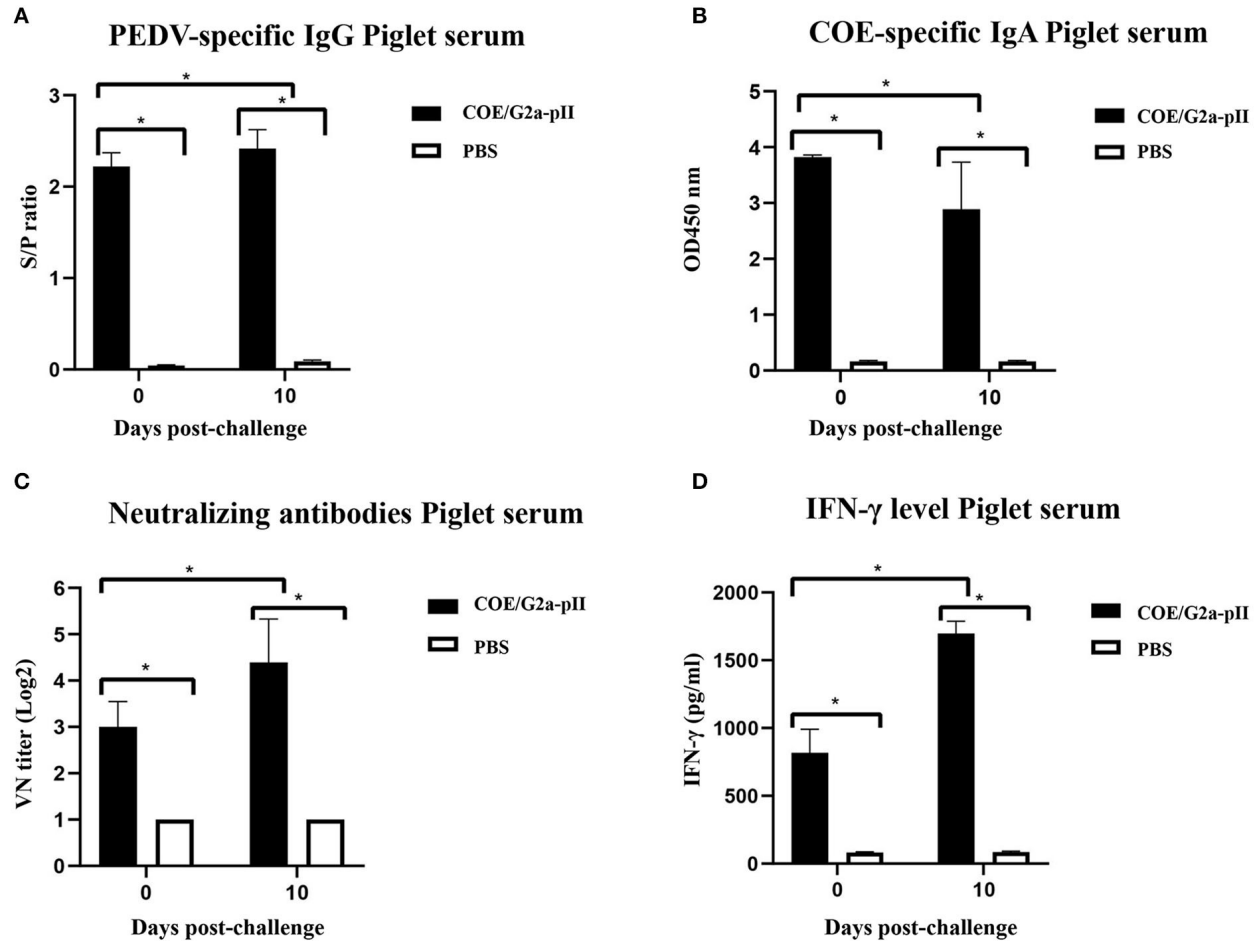
Passive transfer of antibodies and cytokine from sows to piglets at the days 0 and 10 pc. Data are presented as mean ± standard deviation (SD). * indicates a statistically significant difference (*p* < 0.05). **(A)** PEDV-specific IgG antibodies in piglet sera from sows (*n* = 1 per group) vaccinated with COE/G2a-pII, or PBS were measured by a commercial ELISA kit. An S/P ratio > 0.35 was defined as positive with a PEDV-specific IgG antibody. **(B)** COE-specific IgA antibodies in piglet sera (*n* = 8 for COE/G2a-pII group and *n* = 2 for PBS group) were evaluated by an ELISA using SEC-purified COE/G2a-pII as antigen. **(C)** Neutralizing antibodies in piglet sera were analyzed by the virus-neutralizing assay. The highly virulent NAVET/PEDV/PS6/2010 strain (200 TCID50/0.1 ml) was used for the assay. A VN titer ≥ 8 was defined as positive with neutralizing antibody against PEDV. **(D)** IFN-γ levels in piglet sera were measured based on the standard curve of recombinant porcine IFN-γ standard in a commercial ELISA kit.

Since colostrum/milk antibodies from vaccinated mother sows are regularly transferred to piglets *via* suckling, passive immunity to the piglets was assessed by ELISA, virus-neutralizing antibody assay, and cytokine assay. On day 0 pc, PEDV-specific IgG antibodies were detected in the 5-day-old piglets born to sows vaccinated with COE/G2a-pII. The increase in PEDV-specific IgG antibody level was found in piglet sera on day 10 pc (Figure 3A). In addition, the sera of these piglets had high COE-specific IgA antibody responses on day 0 pc. However, there was a slight decrease in COE-specific IgA antibody levels in these piglets on day 10 pc (Figure 3B). Neutralizing antibodies against the highly virulent NAVET/PEDV/PS6/2010 strain were detected in piglets born to sows immunized with COE/G2a-pII protein on day 0 pc. Notably, VN titers found in these piglets on day 10 pc were more than 2-fold higher than those in piglets on day 0 pc (Figure 3C). High levels of IFN-γ were detected in sera of these piglets on day 0 pc with a concentration of 824.5 ± 157.68 pg/ml. Notably, there was a strong increase in IFN-γ levels in piglets born to sows inoculated with COE/G2a-pII protein on day 10 pc (Figure 3D). In contrast, no PEDV-specific IgG, COE-specific IgA, virus-neutralizing antibodies, and cytokine responses were observed in piglets born to sows vaccinated with PBS. These results demonstrate that there was a passive immunity transfer of PEDV-specific IgG, COE-specific-IgA, virus-neutralizing antibodies, and IFN-γ responses from immunized pregnant sows to its piglets *via* sow colostrum/milk.

### COE/G2a-PII protein protects piglets against the highly virulent pedv G2a strain

**Figure 4:**
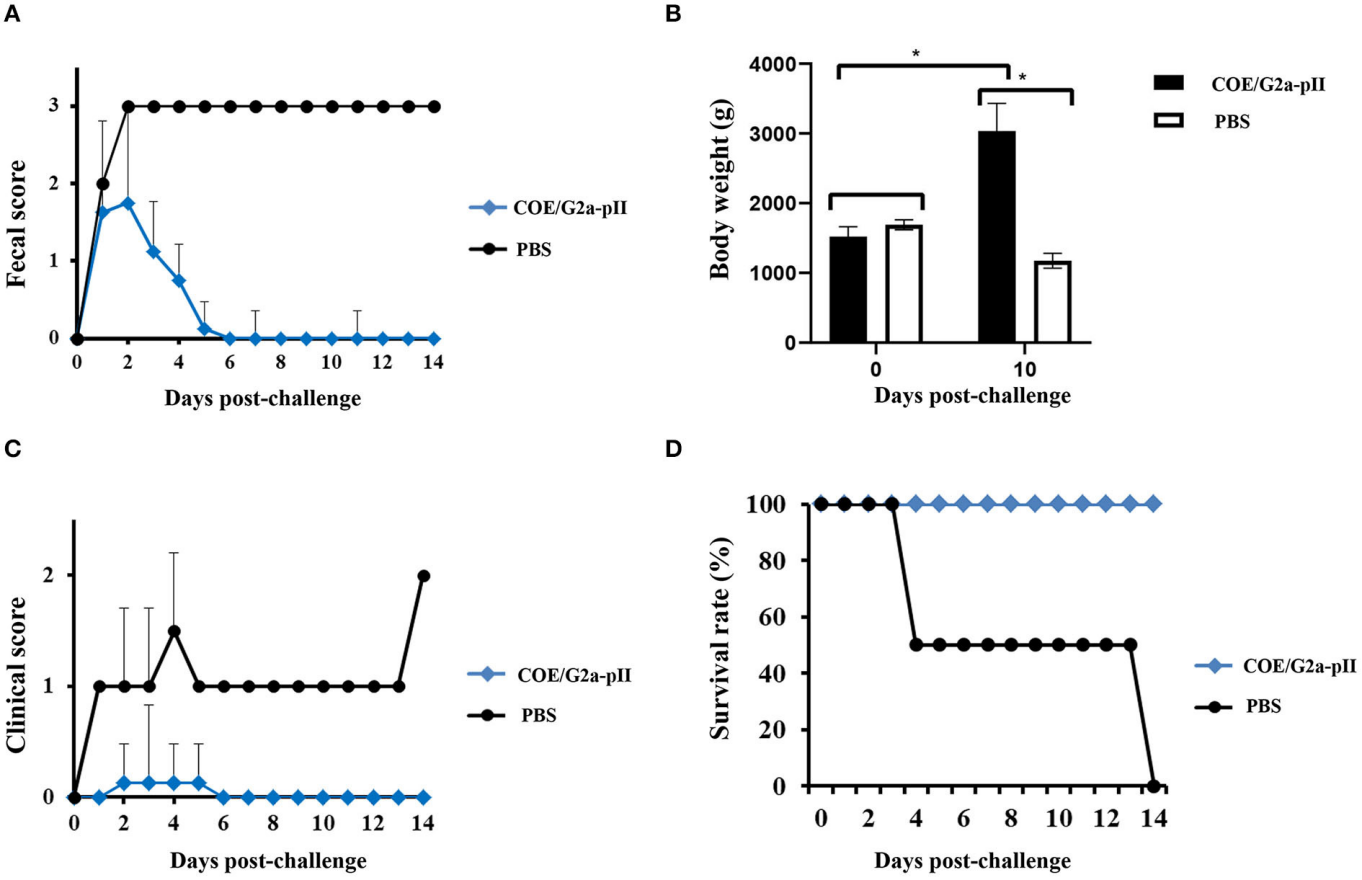
Clinical observation and the survival of piglets obtained passive immunity from sow vaccination. The value is presented as the mean ± standard deviation (SD). * means a statistically significant difference (*p* < 0.05). **(A)** Fecal score of piglets (*n* = 8 for COE/G2a-pII group and *n* = 2 for PBS group) was recorded daily after challenge with highly virulent NAVET/PEDV/PS6/2010 strain. **(B)** The body weight of piglets was calculated on the days 0 and 14 pc. **(C)** The clinical scores of piglets were analyzed daily after the challenge. **(D)** After the challenge, the survival rate of piglets was recorded daily.

After 1 day of the challenge, all piglets born to sows vaccinated with COE/G2a-pII protein had pasty and semisolid feces (fecal scores of 1–2). However, some piglets in this group had normal feces with scores of 0 starting on day 4 pc. All piglets in this group returned to fecal scores of 0 on day 6 pc. In contrast, piglets delivered from sows immunized with PBS had semisolid feces with scores of 2 on day 1 pc. The feces of these piglets were watery, with scores of 3 beginning at day 2 pc to the later (Figure 4A). Before the challenge, there was no significant difference in the body weight of the two piglet groups. After 10 days of the challenge, there was a 2-fold increase in the body weight of piglets delivered from sow inoculated COE/G2a-pII. In contrast, two piglets delivered from negative sow control had a strong decrease in body weight after the challenge compared to that before the challenge (Figure 4B). All piglets born to sows vaccinated with COE/G2a-pII protein had daily normal clinical scores of around 0 and survived after the challenge (Figures 4C,D). In contrast, the clinical signs of piglets delivered from sows immunized with PBS were around 1 (anorectic, depression, vomiting, and emaciated) beginning at day 2 pc, and the clinical scores of this group increased to 1.5 on day 4 pc (Figure 4C). On day 4 pc, one piglet born to the sow vaccinated with PBS died. Another piglet in this group died on day 14 pc (Figure 4D). Therefore, all piglets in the negative control group died after the viral challenge. The cause of the piglet deaths in the negative group was PEDV infections, verified *via* gross lesions in the small intestine of deceased piglets (data not shown). Taken together, these data indicate that passive immunity provided by a pregnant sow vaccinated with COE/G2a-pII protein could induce protective immune responses in its piglets against a highly virulent PEDV G2a strain.

## Discussion

To the best of our knowledge, this is the first study to evaluate the protective efficacy of a plant COE-pII-based subunit vaccine candidate against a highly virulent PEDV G2a in piglets following the passive immunity. Previous studies indicate that the current commercial PEDV vaccine, which contains G1a PEDV strains, may induce low protective immune responses against PEDV strains in the field (9–11). When collecting all DNA sequences encoding the full S protein of Vietnamese PEDV strains published on NCBI, we obtained 63 nucleotide sequences of full S genes. After building the phylogenetic tree based on full S gene sequences of PEDV PS6, 63 Vietnamese strains, and 62 PEDV strains in the world, then comparing the genotype cluster of PEDV strains to previous publications, the Vietnamese PEDV G2a, G2b, and G1b strains were clustered into 35/64 sequences (54.68%), 23/64 sequences (35.93%), 8/64 sequences (12.5%), respectively (data not shown). This finding is also in agreement with the results of previous studies that PEDV strains belonging to the G2 group were detected in most of the PED outbreaks in Vietnam (4). In addition, we also found that PEDV G2a strains were the dominant agents in PED outbreaks in Vietnam. Therefore, the goal of this study was to assess whether immunization with plant-based COE/G2a-pII protein would elicit protective responses in piglets born to immunized sows against highly virulent PEDV G2a strains isolated in Vietnam.

COE/G2a-pII protein was successfully expressed at a high level in plants *via* agroinfiltration. When compared to previous studies, the expression level of COE/G2a-pII protein in *N. benthamiana* is higher than that of COE proteins in previous publications (13, 17, 21, 29). However, the expression level of COE/G2a-pII protein is lower than that of COE/G1a-pII protein in our previous study (234 mg/kg fresh leaves, 4% TSP). This might be explained by the difference in nucleotide sequence and amino acid sequence of the two COE variants. When compared to the native sequence of COE/G1a-pII, the identity in the native nucleotide sequence and amino acid of COE/G2a-pII was 95.7 and 96.4%, respectively (Supplementary Tables 1, 2). After a commercial codon optimization for expression in *N. benthamiana*, the similarity in the nucleotide sequence of two optimized COE variants was 75.5% (Supplementary Table 3). COE/G2a-pII protein is a mixture of multimer forms with a molecular weight of approximately 440 kDa. The size of COE/G2a-pII protein is quite different when compared to our previous publication that used native PAGE to estimate the size of COE/G1a-pII protein (22). This can be explained by the difference in the capacity of each method used to calculate the size of the protein. When compared to SEC, native PAGE has limitations in the separation of high molecular weight protein. Since high mortality caused by PEDV infection occurs mostly in neonatal piglets at the age of under 1 week, an ideal commercial vaccine against PEDV infection should immunize sows to facilitate lactogenic immunity to be transferred to neonatal piglets following suckling. Results presented here indicate that 100% of 5-days-old-piglets born to COE/G2a-pII-vaccinated sows survived after the challenge with a highly virulent PEDV strain belonging to the G2a group. All piglets born to a PBS-vaccinated sow died. The survival rate obtained in piglets of the PBS group agreed with previous studies in which very high mortality (up to 80%−100%) in suckling piglets infected with PEDV at the age of under 7 days (3, 30). The zero mortality rate in piglets born to COE/G2a-pII-vaccinated sow after the challenge can be partly explained by the strong PEDV-specific IgG, COE-specific IgA, neutralizing antibodies, and IFN-γ responses in sow and transferred passively to piglets. The level of neutralizing antibody responses induced in the COE/G2a-pII-vaccinated sow and its piglets was quite similar to those elicited in sows vaccinated with the inactivated PEDV and their offsprings (31). The decrease in the level of humoral immune responses in the serum of sows vaccinated with COE/G2a-PII protein on day 50 pi might be explained by the transportation of antibodies from the blood to the milk of the sow. The IgA, IgG, and neutralizing antibody response in the serum of piglets on day 10 pc was quite similar to those in the serum of sow on day 50 pi. These findings agreed with those in a previous study in which the level of IgG and IgA in piglets mimicked those in sow sera and colostrums (32). In addition, the humoral immune responses induced in sows and piglets could change at the different time points of sampling. Further study should evaluate humoral immune response in sow and piglet sera at several time points.

Cytokines delivered from maternal responses may play important roles in generating the newborn immune response. IFN-γ is a crucial cytokine that is involved in both innate and adaptive immune responses. It has a critical role in the activation of macrophages and the induction of T helper lymphocyte differentiation regarding the Th1, consequent aiding cellular responses and enhancing the protective immune response against pathogen infections (33). Therefore, the evaluation of IFN-γ amount is favored to partially characterize the immune response (34). Notably, the level of IFN-γ in sow (day 35 pi) and its offspring (day 0 pc) were detected at a very high concentration of 932.3 ± 145.2 pg/ml and 824.5 ± 157.68 pg/ml, respectively. When compared to the previous studies, the concentration of IFN-γ in sows vaccinated with COE/G2a-pII at farrowing was over 5-fold higher than that in sows vaccinated with killed-PEDV vaccine plus flagellin protein (Vac201-FliC) (31) and over 10-fold higher than that in sows vaccinated with inactivated PEDV harboring IgG-Fc (35). The level of IFN-γ in sow (day 50 pi) and its offspring (day 10 pc) were 2-fold increased in comparison with those before the challenge.

In this study, the amount of COE/G2a-pII protein used to vaccinate sow was 100 μg per dose, which was 4-fold lower than that used in a previous study (36). The pregnant sow in the previous study was vaccinated three times with polyphosphazene in a triple adjuvant combination (TriAdj) with adjuvanted S1 protein (400 μg) (36). After the viral challenge, the survival rate of piglets delivered from the S1-vaccinated sow in the previous study (36) was 87.5%, which was also lower than the survival rate of our piglets born to COE/G2a-pII sow (100%). The lower required dose of plant-based COE/G2a-pII protein for vaccination resulting in the higher protective efficacy against the highly virulent PEDV G2a can be explained by the formation of multimer forms with high molecular weight in the COE/G2a-pII protein. These results indicate that COE/G2a-pII protein produced in plants could be a potential vaccine candidate to prevent PEDV infection. However, our study still has limitations. The number of sows and piglets used in this study was not large as African swine fever outbreaks in Vietnam seriously affected the pig industry when the animal experiment was performed. The results we achieved prompted us to plan further studies involving many more sows and piglets to validate these data. Moreover, the amount of COE/G2a-pII protein per dose and the effect of different adjuvants that improve immune responses will be addressed in further experiments. In addition, the presence or absence of the PEDV in pig feces after the challenge should also be analyzed.

## Conclusion

The COE/G2a-pII protein produced in plants induced strong humoral immune responses and IFN-γ responses in the pregnant sow. The sow's immune responses were passively transferred to their piglets following suckling. All piglets born to COE/G2a-pII-vaccinated sows survived and had normal clinical signs after the challenge with the highly virulent PEDV G2a strain. These results suggest that the COE/G2a-pII protein is a potential vaccine candidate to prevent PEDV G2a infection of swine in Vietnam, and these preliminary but important data are the basis for performing more extended studies to develop a plant-based COE/G2a-pII vaccine.

## Data availability statement

The original contributions presented in the study are included in the article/Supplementary material, further inquiries can be directed to the corresponding authors.

## Ethics statement

The animal study was reviewed and approved by the Institute of Biotechnology (IBT), Vietnam Academic of Science and Technology (VAST), Hanoi, Vietnam, and NAVETCO, Ho Chi Minh City, Vietnam under decision number 07/2015/HÐ-NÐT.

## Author contributions

HC, NP, and TH designed the study. TH and VT constructed vectors and performed ELISA IgA and IFN-γ. VT and TN performed transient expression. TH, VT, and TN purified protein. HT and PL performed pig immunization and challenge experiments, performed the virus-neutralizing antibody assay, ELISA IgG, and collected the clinical signs of piglets. TH performed the calculations and data analysis and wrote the manuscript. HC, NP, UC, MP, and HH revised the manuscript. HC and NP hold all the responsibilities related to this manuscript. All authors have read and approved the final manuscript.

## Funding

This study was financed by the Ministry of Science and Technology through the project: Development of novel nanoconjugates from nanodiamonds and spike protein of porcine epidemic diarrhea virus toward applications in nanovaccine (code: NT/TW/21/09). This study is a part of the thesis results of Ph.D. student TH at GUST, VAST, Hanoi, Vietnam. TH was funded by Vingroup JSC and supported by the Master, Ph.D. Scholarship Programme of Vingroup Innovation Foundation (VINIF), Institute of Big Data (code: VINIF.2021.TS.066).

## Conflict of interest

The authors declare that the research was conducted in the absence of any commercial or financial relationships that could be construed as a potential conflict of interest.

## Publisher's note

All claims expressed in this article are solely those of the authors and do not necessarily represent those of their affiliated organizations, or those of the publisher, the editors and the reviewers. Any product that may be evaluated in this article, or claim that may be made by its manufacturer, is not guaranteed or endorsed by the publisher.

## References

[1] Song D Moon H Kang B. Porcine epidemic diarrhea: a review of current epidemiology and available vaccines. Clin Exp Vaccine Res. (2015) 4:166–76. 10.7774/cevr.2015.4.2.166 26273575 PMC4524901

[2] Sun RQ Cai RJ Chen YQ Liang PS Chen DK Song CX. Outbreak of porcine epidemic diarrhea in suckling piglets China. Emerg Infect Dis. (2012) 18:161–3. 10.3201/eid1801.111259 22261231 PMC3381683

[3] Stevenson GW Hoang H Schwartz KJ Burrough ER Sun D Madson D. Emergence of Porcine epidemic diarrhea virus in the United States: clinical signs, lesions, and viral genomic sequences. J Vet Diagn Invest. (2013) 25:649–54. 10.1177/1040638713501675 23963154

[4] Than VT Choe SE Vu TTH Do TD Nguyen TL Bui TTN. Genetic characterization of the spike gene of porcine epidemic diarrhea viruses (PEDVs) circulating in Vietnam from 2015 to 2016. Vet Med Sci. (2020) 6:535–42. 10.1002/vms3.256 32159913 PMC7397879

[5] Duy DT Toan NT Puranaveja S Thanawongnuwech R. Genetic characterization of porcine epidemic diarrhea virus (PEDV) isolates from Southern Vietnam during 2009–2010 outbreaks. Thai J Vet Med. (2011) 41:55–64.

[6] Okda FA Lawson S Singrey A Nelson J Hain KS Joshi LR. The S2 glycoprotein subunit of porcine epidemic diarrhea virus contains immunodominant neutralizing epitopes. Virology. (2017) 509:185−94. 10.1016/j.virol.2017.06.013 28647506 PMC7111671

[7] Lee C. Porcine epidemic diarrhea virus: an emerging and reemerging epizootic swine virus. Virol J. (2015) 12:193. 10.1186/s12985-015-0421-2 26689811 PMC4687282

[8] Deng F Ye G Liu Q Navid MT Zhong X Li Y. Identification and comparison of receptor binding characteristics of the spike protein of two porcine epidemic diarrhea virus strains. Viruses. (2016) 8:55. 10.3390/v8030055 26907329 PMC4810246

[9] Diep NV Sueyoshi M Izzati U Fuke N Teh APP Lan NT. Appearance of US-like porcine epidemic diarrhoea virus (PEDV) strains before US outbreaks and genetic heterogeneity of PEDVs collected in Northern Vietnam during 2012–2015. Transbound Emerg Dis. (2018) 65: e83–e93. 10.1111/tbed.12681 28758349 PMC7169849

[10] Li W Li H Liu Y Pan Y Deng F Song Y. New variants of porcine epidemic diarrhea virus China, 2011. Emerg Infect Dis. (2012) 18:1350–3. 10.3201/eid1803.120002 22840964 PMC3414035

[11] Wang X Chen J Shi D Shi H Zhang X Yuan J. Immunogenicity and antigenic relationships among spike proteins of porcine epidemic diarrhea virus subtypes G1 and G2. Arch Virol. (2016) 161:537–47. 10.1007/s00705-015-2694-6 26611909 PMC7087089

[12] Chang SH Bae JL Kang TJ Kim J Chung GH Lim CW. Identification of the epitope region capable of inducing neutralizing antibodies against the porcine epidemic diarrhea virus. Mol Cells. (2002) 14:295–9. 12442904

[13] Huy NX Kim SH Yang MS Kim TG. Immunogenicity of a neutralizing epitope from porcine epidemic diarrhea virus: M cell targeting ligand fusion protein expressed in transgenic rice calli. Plant Cell Rep. (2012) 31:1933−42. 10.1007/s00299-012-1306-0 22736145 PMC7080027

[14] Hajibehzad SS Honari H Nasiri J Mehrizi FA Alizadeh H. High-level transient expression of the N-terminal domain of IpaD from Shigella dysenteriae in four plant species transformed with different construct configurations. In Vitro Cell Dev Biol Plant. (2016) 52:293–302. 10.1007/s11627-016-9760-y

[15] Wroblewski T Tomczak A Michelmore R. Optimization of Agrobacteriummediated transient assays of gene expression in lettuce, tomato and Arabidopsis. Plant Biotechnol J. (2005) 3:259–73. 10.1111/j.1467-7652.2005.00123.x 17173625

[16] Huy NX Kim YS Jun SC Jin Z Park SM Yang MS. Production of a heat-labile enterotoxin B subunit-porcine epidemic diarrhea virus-neutralizing epitope fusion protein in transgenic lettuce (*Lactuca sativa*). Biotechnol Bioprocess Eng BBE. (2009) 14:731–7. 10.1007/s12257-009-3012-5 32218676 PMC7091058

[17] Huy NX Yang MS Kim TG. Expression of a cholera toxin B subunit-neutralizing epitope of the porcine epidemic diarrhea virus fusion gene in transgenic lettuce (*Lactuca sativa* L). Mol Biotechnol. (2011) 48:201–9. 10.1007/s12033-010-9359-1 21153716

[18] Huy NX Tien NQD Kim MY Kim TG Jang YS Yang MS. Immunogenicity of an S1D epitope from porcine epidemic diarrhea virus and cholera toxin B subunit fusion protein transiently expressed in infiltrated Nicotiana benthamiana leaves. Plant Cell Tissue Organ Cult. (2016) 127:369–80. 10.1007/s11240-016-1059-5 32214565 PMC7088629

[19] Tien NQ Huy NX Kim MY. Improved expression of porcine epidemic diarrhea antigen by fusion with cholera toxin B subunit and chloroplast transformation in Nicotiana tabacum. Plant Cell Tissue Organ Cult. (2019) 137:213–23. 10.1007/s11240-019-01562-1 32214566 PMC7089040

[20] Egelkrout E Hayden C Fake G Keener T Arruda P Saltzman R. Oral delivery of maize-produced porcine epidemic diarrhea virus spike protein elicits neutralizing antibodies in pigs. Plant Cell Tissue Organ Cult. (2020) 142:79–86. 10.1007/s11240-020-01835-0 32394992 PMC7212245

[21] Tien NQ Yang MS Jang YS Kwon TH Reljic R Kim MY. Systemic and Oral Immunogenicity of Porcine Epidemic Diarrhea Virus Antigen Fused to Poly-Fc of Immunoglobulin G and Expressed in ΔXT/FT Nicotiana benthamiana Plants. Front Pharmacol. (2021) 12:653064. 10.3389/fphar.2021.653064 33995068 PMC8120289

[22] Ho TT Nguyen GT Pham NB Le VP Trinh TBN Vu TH. Plant-derived trimeric CO-26K-equivalent epitope induced neutralizing antibodies against porcine epidemic diarrhea virus. Front Immunol. (2020) 11:2152. 10.3389/fimmu.2020.02152 33042128 PMC7524870

[23] Pham VD Hoang H Phan HT Conrad U Chu HH. Production of antibody labeled gold nanoparticles for influenza virus H5N1 diagnosis kit development. Adv Nat Sci Nanosci Nanotechnol. (2012) 3:045017. 10.1088/2043-6262/3/4/045017

[24] Phan HT Ho TT Chu HH Vu TH Gresch U Conrad U. Neutralizing immune responses induced by oligomeric H5N1-hemagglutinins from plants. Vet Res. (2017) 48:53. 10.1186/s13567-017-0458-x 28931425 PMC5607582

[25] Hofmann M Wyler R. Quantitation biological and physicochemical properties of cell culture-adapted porcine epidemic diarrhea coronavirus (PEDV). Vet Microbiol. (1989) 20:131–42. 10.1016/0378-1135(89)90036-9 2549681 PMC7117183

[26] Joshi LR Okda FA Singrey A Maggioli MF Faccin TC Fernandes MHV. Passive immunity to porcine epidemic diarrhea virus following immunization of pregnant gilts with a recombinant orf virus vector expressing the spike protein. Arch Virol. (2018) 163:2327–35. 10.1007/s00705-018-3855-1 29725899 PMC7086649

[27] Annamalai T Saif LJ Lu Z Jung K. Age-dependent variation in innate immune responses to porcine epidemic diarrhea virus infection in suckling versus weaned pigs. Vet Immunol Immunopathol. (2015) 168:193–202. 10.1016/j.vetimm.2015.09.006 26433606 PMC7112776

[28] Kusanagi K Kuwahara H Katoh T Nunoya T Ishikawa Y Samejima T. Isolation and serial propagation of porcine epidemic diarrhea virus in cell cultures and partial characterization of the isolate. J Vet Med Sci. (1992) 54:313–8. 10.1292/jvms.54.313 1318752

[29] Kang TJ Kim YS Jang YS. Expression of the synthetic neutralizing epitope gene of porcine epidemic diarrhea virus in tobacco plants without nicotine. Vaccine. (2005) 23:2294–7. 10.1016/j.vaccine.2005.01.027 15755614

[30] Carvajal A Argüello H Martínez-Lobo FJ et al. Porcine epidemic diarrhoea: new insights into an old disease. Porc Health Manag. (2015) 1:12. 10.1186/s40813-015-0007-9 28405418 PMC5382377

[31] Xu X Du L Fan B. A flagellin-adjuvanted inactivated porcine epidemic diarrhea virus (PEDV) vaccine provides enhanced immune protection against PEDV challenge in piglets. Arch Virol. (2020) 165:1299–309. 10.1007/s00705-020-04567-w 32253616 PMC7223252

[32] Bandrick M Ariza-Nieto C Baidoo SK Molitor TW. Colostral antibody-mediated and cell-mediated immunity contributes to innate and antigen-specific immunity in piglets. Dev Comp Immunol. (2014) 43:114–20. 10.1016/j.dci.2013.11.005 24252519 PMC3902642

[33] Kumar V Abbas AK Aster JC. Robbins & Cotran Pathologic Basis of Disease, 10th Edn. Philadelphia, PA: El Sevier (2020).

[34] Navarro E Mainau E de Miguel R Temple D Salas M Manteca X. Oral meloxicam administration in sows at farrowing and its effects on piglet immunity transfer and growth. Front Vet Sci. (2021) 8:574250. 10.3389/fvets.2021.574250 33681319 PMC7928392

[35] Park JE Jang H Kim J-H Hyun B-H Shin H-J. Immunization with porcine epidemic diarrhea virus harbouring Fc domain of IgG enhances antibody production in pigs. Vet Q. (2020) 40:183–9. 10.1080/01652176.2020.1773006 32448096 PMC7734062

[36] Makadiya N Brownlie R van den Hurk J Berube N Allan B Gerdts V. S1 domain of the porcine epidemic diarrhea virus spike protein as a vaccine antigen. Virol J. (2016) 13:57. 10.1186/s12985-016-0512-8 27036203 PMC4818391

