# Supplementary material for: The immunogenicity of plant-based COE-GCN4pII protein in pigs against the highly virulent porcine epidemic diarrhea virus strain from genotype 2
Source: Front Vet Sci. 2022 Jul 28;9:940395. doi: 10.3389/fvets.2022.940395 (PMC9366249; doi:10.3389/fvets.2022.940395)
Supplement: Supplementary file 1 [file Data_Sheet_1.docx]

**SUPPLEMENTAL DATA**

**Table S1. Sequence comparison and pair distances of native nucleotide sequences encoding two COE variants from two PEDV strains.**

10 20 30 40 50 60 70 80 90 100

....|....|....|....|....|....|....|....|....|....|....|....|....|....|....|....|....|....|....|....|

**COE-NAVET-PS6-2010-G2a** **GTTACTTTGCCATCATTTAATGATCATTCTTTTGTTAATATTACTGTCTCTGCTGCTTTTGGTGGTCATAGTGGTGCCAACCTTATTGCATCTGACACTA**

**COE-DR13-G1a**  **.................C...................................G.............................C................**

110 120 130 140 150 160 170 180 190 200

....|....|....|....|....|....|....|....|....|....|....|....|....|....|....|....|....|....|....|....|

**COE-NAVET-PS6-2010-G2a** **CTATCAATGGGTTTAGTTCTTTCTGTGTTGACACTAGACAATTTACCATTTCACTGTTTTATAACGTTACAAACAGTTATGGTTATGTGTCTAAATCACA**

**COE-DR13-G1a**  **..................................................A...........................................G.....**

210 220 230 240 250 260 270 280 290 300

....|....|....|....|....|....|....|....|....|....|....|....|....|....|....|....|....|....|....|....|

**COE-NAVET-PS6-2010-G2a** **AGACAGTAATTGCCCTTTTACCTTGCAATCTGTTAATGATTACCTGTCTTTTAGCAAATTTTGTGTTTCTACCAGCCTTTTGGCTAGTGCCTGTACCATA**

**COE-DR13-G1a**  **G..T..............C..................................................A...............G....T.........**

310 320 330 340 350 360 370 380 390 400

....|....|....|....|....|....|....|....|....|....|....|....|....|....|....|....|....|....|....|....|

**COE-NAVET-PS6-2010-G2a** **GATCTTTTTGGTTACCCTGAGTTTGGTAGTAGTGTTAAGTTTACGTCCCTCTACTTTCAATTCACAAAGGGTGAGTTGATTACTGGCACGCCTAAACCAT**

**COE-DR13-G1a**  **.......................C......G...................T..T.............................................C**

410 420

....|....|....|....|

**COE-NAVET-PS6-2010-G2a** **TTGAAGGTGTTACGGACGTT**

**COE-DR13-G1a**  **..C.......C.........**


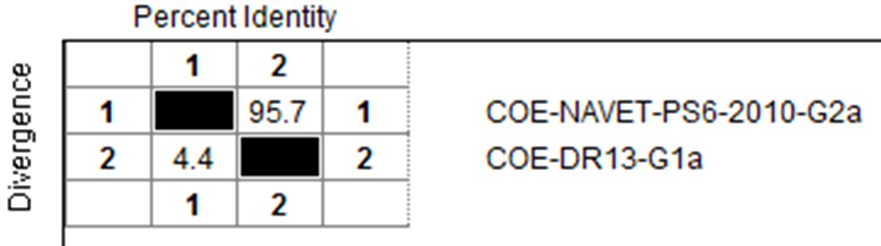


Two native nucleotide sequence encoding COE proteins of PEDV strains of the highly virulent NAVET/PEDV/PS6/2010 strain and PEDV/attenuated DR13 were aligned by using clustal W method in DNASTAR software (Lasergene). As presented in Table S1, COE from the highly virulent NAVET/PEDV/PS6/2010 strain had 95.7% nucleotide sequence similarity with that from PEDV/attenuated DR13. The the highly virulent NAVET/PEDV/PS6/2010 strain and PEDV/attenuated DR13 were classified into the G2a and G1a, respectively.

**Table S2. Sequence comparison and pair distances of amino acid sequences encoding two COE variants from two PEDV strains.**

10 20 30 40 50 60 70 80 90 100

....|....|....|....|....|....|....|....|....|....|....|....|....|....|....|....|....|....|....|....|

**COE-NAVET-PS6-2010-G2a** **VTLPSFNDHSFVNITVSAAFGGHSGANLIASDTTINGFSSFCVDTRQFTISLFYNVTNSYGYVSKSQDSNCPFTLQSVNDYLSFSKFCVSTSLLASACTI**

**COE-DR13-G1a**  **..................................................T............................................G....**

110 120 130 140

....|....|....|....|....|....|....|....|

**COE-NAVET-PS6-2010-G2a** **DLFGYPEFGSSVKFTSLYFQFTKGELITGTPKPFEGVTDV**

**COE-DR13-G1a**  **..........G......................LQ.....**

**
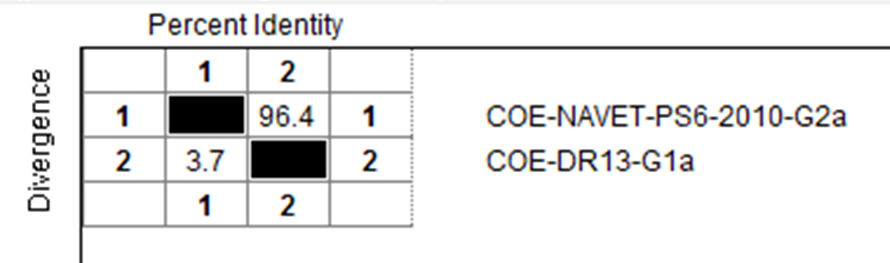
**

Two optimized amino acid sequences of deduced COE proteins of PEDV strains of the highly virulent NAVET/PEDV/PS6/2010 strain and PEDV/attenuated DR13 for expression in *N. benthamiana* were aligned by using clustal W method in DNASTAR software (Lasergene). As presented in Table S3, COE from the highly virulent NAVET/PEDV/PS6/2010 strain had 96.4% amino acid sequence similarity with that from PEDV/attenuated DR13.

**Table S3. Sequence comparison and pair distances of optimized nucleotide sequences encoding two COE variants for expression in *N. benthamiana***

....|....|....|....|....|....|....|....|....|....|....|....|....|....|....|....|....|....|....|....|

**Optimized COE/NAVET/PS6/G2a** **GTGACTCTGCCTTCTTTCAACGATCACTCCTTCGTGAACATCACCGTGTCTGCTGCTTTCGGTGGTCATTCTGGTGCTAACCTTATCGCTTCCGACACCA**

**Optimized COE/DR13/G1a**  **......T.....AG.........C...AGT..T..T.....T..A..T..A..A..C...........C.....A.....T.....A.....T.....T.**

110 120 130 140 150 160 170 180 190 200

....|....|....|....|....|....|....|....|....|....|....|....|....|....|....|....|....|....|....|....|

**Optimized COE/NAVET/PS6/G2a** **CTATCAACGGCTTCAGCTCTTTCTGTGTGGACACCAGGCAGTTCACCATCAGCCTGTTCTACAACGTGACCAACAGCTACGGCTACGTGAGCAAGTCTCA**

**Optimized COE/DR13/G1a**  **.C..T.....A...TCA........C..T..T........A.....T....CT..T.....T.....A.....TTCT...........CTCT...AG...**

210 220 230 240 250 260 270 280 290 300

....|....|....|....|....|....|....|....|....|....|....|....|....|....|....|....|....|....|....|....|

**Optimized COE/NAVET/PS6/G2a** **GGATTCTAACTGCCCGTTCACCTTGCAGTCCGTGAACGATTACCTGAGCTTCAGCAAGTTCTGCGTGTCAACCTCTCTTCTGGCTTCTGCTTGCACCATC**

**Optimized COE/DR13/G1a**  **A...........T..A.....AC.T..A..T.....T..C.....TTCT...TCA.....T..T...........A...T.....GGA..C.....T..T**

310 320 330 340 350 360 370 380 390 400

....|....|....|....|....|....|....|....|....|....|....|....|....|....|....|....|....|....|....|....|

**Optimized COE/NAVET/PS6/G2a** **GATCTGTTTGGTTACCCTGAGTTCGGCAGCTCTGTGAAGTTCACCTCACTGTACTTCCAGTTCACTAAGGGCGAGCTGATTACTGGAACCCCTAAGCCTT**

**Optimized COE/DR13/G1a**  **...T.......A..T..A..A......TCTGGA..T.....T..T..T..T...........T...........AT....C.....C..T........A.**

410 420

....|....|....|....|

**Optimized COE/NAVET/PS6/G2a** **TTGAGGGTGTGACCGATGTG**

**Optimized COE/DR13/G1a**  **.GC.A..A.....A.....T**

**
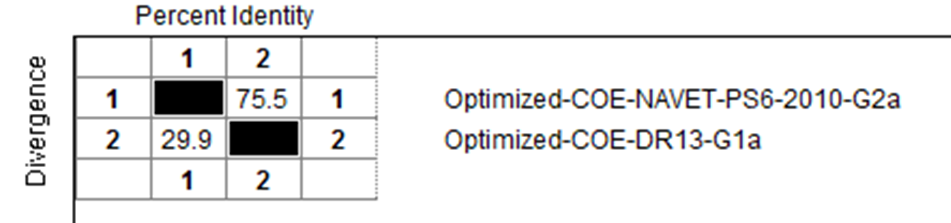
**

Two optimized nucleotide sequences encoding COE proteins of PEDV strains of the highly virulent NAVET/PEDV/PS6/2010 strain and PEDV/attenuated DR13 for expression in *N. benthamiana* were aligned by using clustal W method in DNASTAR software (Lasergene). As presented in Table S2, COE from the highly virulent NAVET/PEDV/PS6/2010 strain had 75.5% nucleotide sequence similarity with that from PEDV/attenuated DR13.
